# Femoral Access for Central Venous Port System Implantation

**DOI:** 10.7759/cureus.2327

**Published:** 2018-03-14

**Authors:** Mikhail Cherkashin, Natalia Berezina, Denis Puchkov, Kirill Suprun, Petr Yablonsky

**Affiliations:** 1 Surgery, Dr Berezin Medical Institute; 2 Radiology, Dr Berezin Medical Institute; 3 Thoracic Surgery, Saint-Petersburg State University

**Keywords:** venous thromboembolism, medical oncology, patient outcome, vascular access port, minimal access surgical procedures

## Abstract

Totally implanted venous access port (TIVAP) systems provide adequate quality of care and life, especially for oncology patients. Long-term vascular access is very important and easy to perform, but in some clinical situations, if patients have a superior caval system occlusion, femoral insertion may be the only option. We present a case of a 70-year-old colorectal adenocarcinoma patient diagnosed with subclavian vein hypoplasia. Her care team decided intraoperatively to implant a port system by the right femoral access. The patient was included in an active surveillance program with regular follow-ups and has had no complications by the end of 2017. This uncommon surgical approach provided this cancer patient with an opportunity to realize a chemotherapy program with optimal quality of life.

## Introduction

Totally implanted venous access port (TIVAP) systems are an important part of modern medical oncology care [1]. Intravenous ports not only provide adequate vascular access for medical needs, but they also reduce the frequency of vessel punctures and improve patient quality of care and life in general [2].

Device insertion via subclavian or jugular access requires imaging control (ultrasound or X-ray) to direct the catheter. This procedure is relatively easy to perform and is not complicated. Sometimes an electrocardiography probe for catheter tip positioning is useful in addition to imaging modalities. However, in superior caval system occlusions, a radiologist or surgeon needs to find an alternative access for catheter insertion, such as via the femoral vein. Femoral TIVAP placement is a rare intervention; Goltz et al. [3] reported an incidence rate of just 0.47% for this entry access. The indications for femoral access are currently unclear. Femoral access carries an expected higher thrombogenicity for devices, and surgeons typically try to avoid alternative vascular entries. Piran et al. [2] reported an 8.5% incidence of venous thromboembolism (VTE) in patients with subclavian TIVAP. However, the femoral entry may be associated with stronger endothelial injury, lower blood flow velocity, and, in theory, a higher rate of complications. Guidelines for routine anticoagulant use for VTE prevention for patients with implanted port systems are currently unclear. Based on the results of small observation trials, most clinicians decide to avoid the use of anticoagulants [2,4].

## Case presentation

A 70-year-old woman presented with metastatic colorectal adenocarcinoma. She had a primary hemicolectomy in 2013 with local recurrence and secondary surgery and liver metastases treated with radiofrequency ablation in 2016. A computed tomography (CT) scan of her chest revealed the progression of her disease via multiple lung metastases in March 2017. The patient had a history of deep vein thrombosis three months earlier (right calf) and received rivaroxaban with good clinical and ultrasound outcome. She was admitted to our hospital for TIVAP insertion and polychemotherapy initiation.

Surgery was performed under local anesthesia (April 2017). Initially, the right subclavian blind cannulation by the surgeon was ineffective. An experienced anesthesiologist was invited into the operating room, but multiple ultrasound-guided cannulation attempts failed bilaterally. The patient was transferred to the radiology department, and a chest CT scan revealed subclavian vein hypoplasia (vessel diameter was up to 4 mm). We decided to use right common femoral venous entry and discussed the situation with the patient. The patient provided additional informed consent to proceed. A catheter was inserted via femoral access without complications. In the operating room, the venous catheter was replaced by tubing from the port system kit.

Catheter tip positioning was performed using transabdominal ultrasound guidance (with a convex probe). The capsule was implanted subcutaneously on the front lateral side of the right hip. Postoperatively, a whole-body CT scan allowed us to assess the TIVAP position and identify possible complications. Technical success was confirmed, and the patient was discharged from the surgery department. Anticoagulation therapy with rivaroxaban was prolonged to avoid a thrombosis recurrence.

As a routine practice, we include all patients with implanted ports in the observational venous thromboembolism monitoring program. This patient visited our hospital for follow-up evaluations one, three, and six months after surgery (May, July, October 2017, respectively). A physical examination showed no signs of venous thrombosis or port system occlusion.

**Figure 1:**
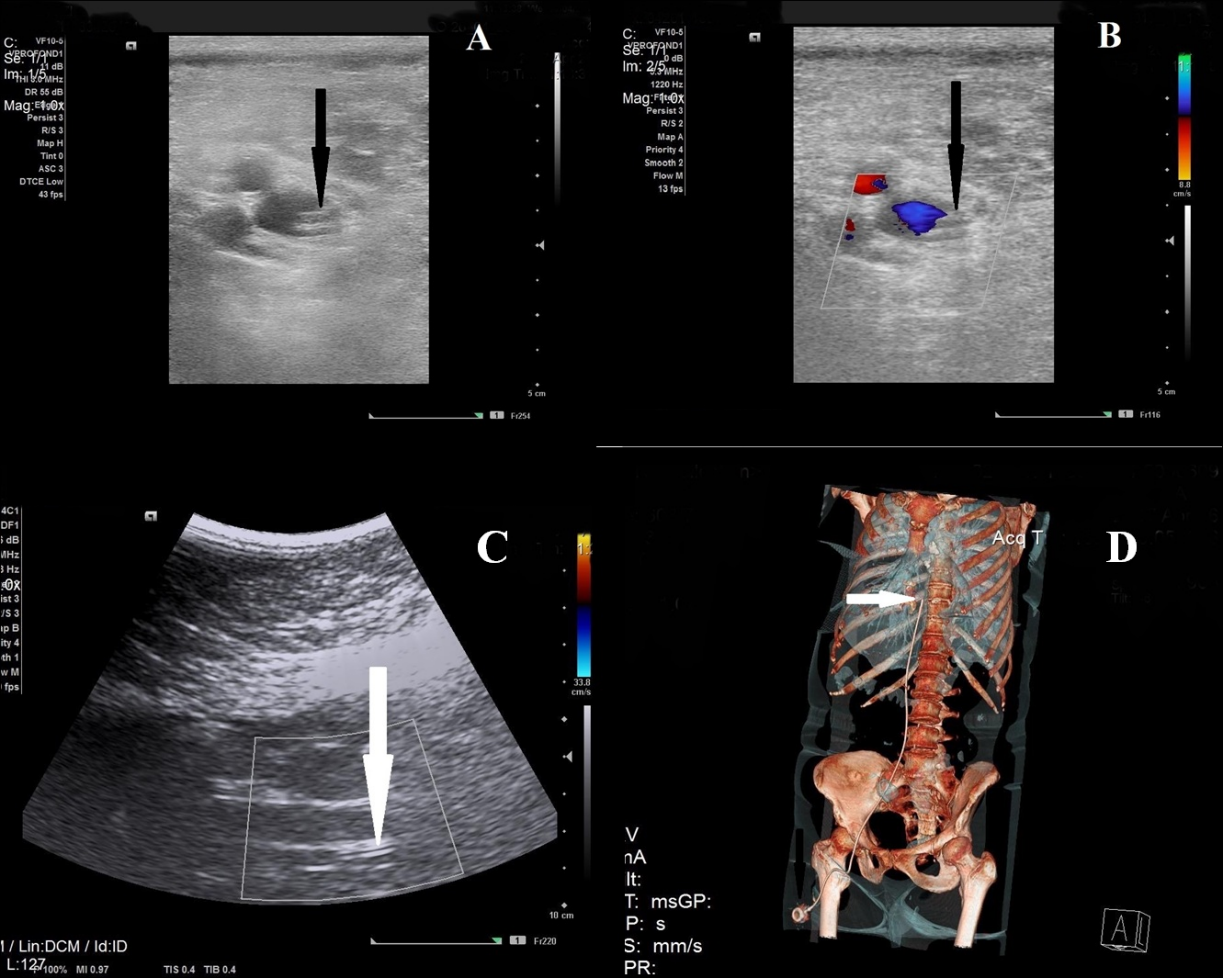
Femoral port-system vizualisation by different imaging modalities A. Ultrasound scan. Saphenofemoral confluence. B-mode, adequate catheter position (arrow). B. Ultrasound scan. Saphenofemoral confluence. Color Doppler, adequate catheter position (arrow). C. Transabdominal ultrasound scan. Inferior caval vein. Adequate catheter position (arrow). D. Whole body CT scan. TIVAP capsule on the right hip, catheter tip in the proximal part of IVC close to heart right atrium (white arrow). Abbreviations: CT, computed tomography; TIVAP, totally implanted venous access port; IVC, inferior vena cava.

Vascular ultrasound B-mode and color Doppler examination were performed (Figures 1A, 1B, 1C). No ultrasound signs of thrombosis, fibrin deposits, catheter occlusion, or perforation were detected. A whole-body CT scan (Figure 1D) revealed adequate device position. The patient is currently receiving polychemotherapy in an outpatient setting, and the port system is in use weekly without difficulties (last control - October 2017, next visit estimated in April 2018).

## Discussion

In general, long-term vascular access is characterized by two main controversial questions - thrombotic complications and potential infection. Based on the American Society of Clinical Oncology Guidelines and Khorana VTE risk score, current time TIVAP doesn't demand routine anticoagulant prevention, but in selected patients (for example, with deep vein thrombosis (DVT) history) it is obvious to use conventional drugs on-label to avoid thrombosis recurrence (like rivaroxaban in this case). Port-system infection potentially may be devastating due to sepsis, and patient education is critical.

Femoral entry for TIVAP implantation should be considered case by case after a strong risk-benefit assessment. For example, Chen et al. [5] proposed bilateral breast cancer as an indication for femoral TIVAP. The standard subclavian or jugular cannulation with non-standard capsule placement (on the lateral chest wall or shoulder) looks preferable. Based on our experience and what is reported in the literature [5] we suggest a slightly restricted approach using vascular occlusion alone (extravasal compression, thrombosis or post-thrombotic syndrome, hypoplasia or anatomic variations) to justify non-standard access.

## Conclusions

The main indication for femoral port placement is occlusion of the subclavian vein. Femoral access is easy to perform without technical difficulties but may be a risk factor for thrombotic complications. Routine VTE prophylaxis use is unclear, but if the patient has a history of deep vein thrombosis, anticoagulant use may be a reasonable solution. An active surveillance program (i.e., postoperative monitoring) provides physicians with the opportunity for the timely identification and treatment of early and late complications such as venous thromboembolism.
